# MiR-223 enhances lipophagy by suppressing CTSB in microglia following lysolecithin-induced demyelination in mice

**DOI:** 10.1186/s12944-024-02185-y

**Published:** 2024-06-22

**Authors:** Hao Ma, Zhi-lin Ou, Nima Alaeiilkhchi, Yong-quan Cheng, Kai Chen, Jia-yu Chen, Ru-qin Guo, Min-yue He, Shi-yi Tang, Xin Zhang, Zhi-ping Huang, Junhao Liu, Jie Liu, Qing-an Zhu, Zu-cheng Huang, Hui Jiang

**Affiliations:** 1 Division of Spine Surgery, Department of Orthopaedics, Nanfang Hospital, Southern Medical University, Guangzhou, 510515 Guangdong China; 2 International Collaboration on Repair Discoveries (ICORD), Blusson Spinal Cord Centre, University of British Columbia, Vancouver, Canada; 3 The First School of Clinical Medicine, Nanfang Hospital, Southern Medical University, Guangzhou, 510515 Guangdong China; 4 School of Traditional Chinese Medicine, Southern Medical University, Guangzhou, 510515 Guangdong China; 5 Department of Laboratory Medicine, Nanfang Hospital, Southern Medical University, Guangzhou, 510515 China; 6 Division of Spine Surgery, Department of Orthopaedics, Guangzhou First People’s Hospital, School of Medicine, South China University of Technology, Guangzhou, 51000 China; 7 The 3rd Affiliated Hospital of Xinxiang Medical University, Henan, China

**Keywords:** Demyelination, MiR-223, Lipid droplets, Lipophagy, Microglia, CTSB (cathepsin B)

## Abstract

**Background:**

Lipid droplet (LD)-laden microglia is a key pathological hallmark of multiple sclerosis. The recent discovery of this novel microglial subtype, lipid-droplet-accumulating microglia (LDAM), is notable for increased inflammatory factor secretion and diminished phagocytic capability. Lipophagy, the autophagy-mediated selective degradation of LDs, plays a critical role in this context. This study investigated the involvement of microRNAs (miRNAs) in lipophagy during demyelinating diseases, assessed their capacity to modulate LDAM subtypes, and elucidated the potential underlying mechanisms involved.

**Methods:**

C57BL/6 mice were used for in vivo experiments. Two weeks post demyelination induction at cervical level 4 (C4), histological assessments and confocal imaging were performed to examine LD accumulation in microglia within the lesion site. Autophagic changes were observed using transmission electron microscopy. miRNA and mRNA multi-omics analyses identified differentially expressed miRNAs and mRNAs under demyelinating conditions and the related autophagy target genes. The role of miR-223 in lipophagy under these conditions was specifically explored. In vitro studies, including miR-223 upregulation in BV2 cells via lentiviral infection, validated the bioinformatics findings. Immunofluorescence staining was used to measure LD accumulation, autophagy levels, target gene expression, and inflammatory mediator levels to elucidate the mechanisms of action of miR-223 in LDAM.

**Results:**

Oil Red O staining and confocal imaging revealed substantial LD accumulation in the demyelinated spinal cord. Transmission electron microscopy revealed increased numbers of autophagic vacuoles at the injury site. Multi-omics analysis revealed miR-223 as a crucial regulatory gene in lipophagy during demyelination. It was identified that cathepsin B (CTSB) targets miR-223 in autophagy to integrate miRNA, mRNA, and autophagy gene databases. In vitro, miR-223 upregulation suppressed CTSB expression in BV2 cells, augmented autophagy, alleviated LD accumulation, and decreased the expression of the inflammatory mediator IL-1β.

**Conclusion:**

These findings indicate that miR-223 plays a pivotal role in lipophagy under demyelinating conditions. By inhibiting CTSB, miR-223 promotes selective LD degradation, thereby reducing the lipid burden and inflammatory phenotype in LDAM. This study broadens the understanding of the molecular mechanisms of lipophagy and proposes lipophagy induction as a potential therapeutic approach to mitigate inflammatory responses in demyelinating diseases.

**Supplementary Information:**

The online version contains supplementary material available at 10.1186/s12944-024-02185-y.

## Introduction

Multiple sclerosis (MS) is a demyelinating disease characterized by central nervous system inflammation, resulting in impaired nerve impulse transmission due to loss of the axonal myelin sheath, accompanied by neurodegeneration [1]. In MS, myelin phospholipids and neuronal fragments cause neuroinflammation, further exacerbating demyelination [2, 3]. Microglia, as resident phagocytes of the central nervous system, play a fundamental role in the clearance of myelin debris [4]. Microglia facilitate the remyelination process by providing nutritional support to oligodendrocytes [4]. However, in MS, the capacity of microglia for clearance is limited [5]. The continuous internalization of myelin debris overburdens microglia, leading to the accumulation of numerous LDs within these cells. These LD-rich microglia, also known as lipid-droplet-accumulating microglia (LDAM), exhibit unique transcriptional characteristics associated with cellular dysfunction. These cells exhibit significant phagocytic defects, and increased lipid storage. These LDAM activities hinder remyelination, resulting in neurofunctional impairment [6–8]. Modulating LDAM can thus prevent inflammatory lipid distribution and improve neural function [9].

Autophagy within microglia regulates lipid homeostasis. However, impaired autophagy results in LD accumulation and metabolic dysfunction [10]. Lipophagy, a specific type of autophagy that selectively degrades LDs, is a critical process in this context [11]. In lipophagy, LDs are targeted to lysosomes through ubiquitination, selective autophagy factors, and receptors. They are then hydrolyzed in the lysosomal lumen by lysosomal acid lipase (LAL), producing free cholesterol and fatty acids. These byproducts are either exported from the cell or utilized for fatty acid oxidation in mitochondria [12–15]. Recent studies have shown that targeting lipophagy in microglia can ameliorate the inflammatory milieu in multiple sclerosis and promote remyelination [16], suggesting that lipophagy induction is a promising therapeutic strategy.

MicroRNAs (miRNAs) are pivotal in various cellular biological processes. For instance, miR-223, an evolutionarily conserved anti-inflammatory microRNA, inhibits the proinflammatory phenotype in macrophages and neutrophils, thereby suppressing innate immune system activation [17]. Furthermore, miRNAs that regulate autophagy are emerging as therapeutic targets for diverse diseases [18–21]. MiR-214, a key posttranscriptional regulator of hepatic lipid metabolism, inhibits hepatic cell lipophagy, leading to LD accumulation [22]. In the development of atherosclerotic lipid plaques, miRNA regulation of autophagy-related genes is crucial. For instance, miRNA-29a inhibits plaque formation via PI3K/AKT/mTOR pathway-mediated macrophage autophagy [23]. Early in atherosclerotic plaque development, miRNA-155 targeting promotes autophagy, potentially alleviating atherosclerosis in ApoE -/- mice [24]. However, reports on miRNAs as regulators of lipophagy in neurological diseases are scarce. Exploring the impact of miRNAs on lipophagy in neurological diseases could theoretically enhance the understanding of lipophagy mechanisms and provide novel approaches for promoting LD degradation in LDAM and remyelination in multiple sclerosis.

In this study, LDAM and lipophagy were examined in demyelinated tissues using histology staining and transmission electron microscopy. The key miRNAs and their targets involved in lipophagy during demyelination were identified through a combined multi-omics analysis. This study presents a novel finding that miR-223 facilitates the degradation of lipid droplets (LDs) through lipid phagocytosis following demyelination. This research identifies, for the first time, cathepsin B (Ctsb) as a target for this activity. This study was conducted to provide insights into the miRNAs associated with lipophagy and their regulatory mechanisms in lysolecithin (LPC) induced demyelination.

## Methods and materials

### Animals

Southern Medical University’s Experimental Animal Center provided adult male C57BL/6 mice weighing 18–22 g for this study, which was approved by Nanfang Hospital’s Laboratory Animal Care and Use Committee.

### LPC-induced demyelination animal model

Animals were randomly assigned to one of two experimental groups, the demyelinating (DM) and the Sham group, with 12 mice per group. Demyelination was established through the microinjection of LPC. The procedure was as follows. Isoflurane was used for anesthesia (3% induction, 2% maintenance). The C4 lamina was surgically exposed to access the spinal cord. A stereotactic frame was then installed, and a 5 µL Hamilton microinjection pump was connected. The needle, aligned parallel to the coronal plane, was positioned 0.15 mm to the left of the midline. A 1% lysolecithin solution (1 µL) was injected at a depth of 0.5 mm.

### Tissue preparation

For histological analysis, pentobarbital sodium (80 mg/kg) was used as a deep anesthetic, followed by rapid perfusion with phosphate-buffered saline (PBS) and 4%paraformaldehyde (PFA) perfusion. The spinal cord surrounding the injection site (1 cm in length) was excised postperfusion. Subsequently, the tissues were dehydrated using a sucrose gradient (12%, 18%, 24%), embedded in an OCT compound, and frozen. Cross-sectional slices of the spinal cord were then prepared using a cryostat microtome.

For the miRNA and mRNA microarray analyses, four mice per group were subjected to transcardial perfusion with 20 ml of chilled PBS. Subsequently, a segment approximately 1 cm in length from the fourth cervical spinal cord was excised on ice. These samples were promptly labeled and then immediately stored at − 80 °C.

### Eriochrome cyanine (EC) staining

The slices were washed with xylene to remove the OCT complex and rehydrated with ethanol. The ethanol was then removed with water. Then the slices were placed in the EC solution for 6 min and then rinsed twice with water. Finally, the slices were differentiated with NH4OH: H2O (1:6) and covered with neutral gum.

### Differential expression analysis

The raw data were quantitatively normalized and then differentially expressed genes (DEGs) between demyelinated (DM) and sham samples were identified. Bioconductor4.1 in the “limma” R package was used for normalization and subsequent data processing. The significance threshold for genes was set at an absolute log fold change surpassing 1 alongside a *P*-value below 0.05.

### Gene ontology (GO) and Kyoto encyclopedia of genes and genomes (KEGG) pathway enrichment analyses

GO is a bioinformatics tool that annotates genes, gene products, and sequences using specific terms [19]. KEGG is a database for linking genomic information to higher-order functional information and pathways [21]. The ‘clusterProfiler’ V3.8, a Bioconductor-dependent R package, was employed for automated biological-term classification and enrichment analysis of gene clusters [22]. ClusterProfiler was used to analyze GO and KEGG enrichment of DEGs identified in this study.

### Histopathological examination and immunofluorescence staining (IF)

LDs were detected by staining spinal cord slices with Oil Red O (Solarbio G1262, Beijing, China). Primary antibodies underwent overnight incubation at 4 °C with tissue sections following three washes with PBS for ten minutes each (anti-Iba-1 (Santa Cruz sc-32,725, Heidelberg, Germany) and anti-ASC (Cell Signaling Technology 67,824 S, Danvers, MA, USA)). After rinsing, the sections were incubated for two hours at room temperature with the following fluorescent secondary antibodies: Alexa Fluor 488 (Abcam ab150105, Cambridge, UK) and Alexa Fluor 555 (Abcam ab150062, Cambridge, UK). For LD staining, the sections were incubated with BODIPY for 20 min after secondary antibody incubation, following three washes in PBS, DAPI was used to stain the nuclei.

Cells were first fixed with 4% PFA for 10 min. Subsequently, they underwent three washes in PBS before being subjected to a 12-hour incubation period at 4 °C with anti-LC3 (Proteintech 14600-1-AP, Wuhan, China), anti-IL-1β (Proteintech 16806-1-AP, Wuhan, China), and anti-CTSB (Proteintech 12216-1-AP, Wuhan, China) antibodies. Incubation was followed by two hours of secondary antibody incubation, stained with BODIPY 558/568 C12 (Invitrogen D3835, California, USA) for 20 min, following three washes in PBS, DAPI was used to stain the nuclei.

### Transmission electron microscopy (TEM)

After obtaining spinal cord tissue as described above for tissue preparation, it was postfixed with 2% glutaraldehyde overnight at 4 °C, rinsed with PBS and incubated with osmium tetroxide for 1 h. After successive PBS rinses and dehydration to increase ethanol concentration, samples were permeabilized in acetone-Epon mixtures and embedded in pure Epon overnight. The prepared samples were sent to Baikandu Company for TEM observation and analysis.

### Cell culture and lentiviral transfection

Shanghai Cell Research Center in China provided the murine BV2 microglial cell line. At approximately 80% confluence, the cells were trypsinized and passaged. PSLenti-EF1-mCherry-P2A-Puro-CMV-Mir223-WPRE (miR-223 OE) and a control lentivirus lacking miR-223 (miR-223 NC) were constructed using lentiviral vectors (Heyuan, Shanghai, China). Infected cells were multiplied by these vectors to 40–50% confluency.

### Quantitative real-time PCR

The Viral RNA Purification Kit (EZB-VRN1, Jiangsu, China) was utilized to extract RNA. For reverse transcription and real-time PCR of miR-223, the EZB-miRT2-plus (EZB, Jiangsu, China) and EZB-miProbe-R2 (EZB, Jiangsu, China) were used. The miRNA-223-specific forward primer (GCCCGfCCAGUUUGUCAAAUA) and reverse primer (GTGCAGGGTCCGAGGT) were purchased from Heyuan (Shanghai, China). The reverse transcription of total RNA from *Ctsb* and IL-1β into first-strand cDNA was accomplished using the RT‒PCR Kit (Vazyme P611‒01, Nanjing, China). The quantification of the resultant cDNA transcripts was executed using an SYBR Green Master Mix(Vazyme Q141-02, Nanjing, China) with the following primer sequences: mouse *Ctsb_Forward* primer: 5′-TCCTTGATCCTTCTTTCTTGCC-3′, mouse *Ctsb_Reverse primer*: 5′-ACAGTGCCACACAGCTTCTTC-3′, mouse *IL-1β_ Forward*: 5′-AAGATGAAGGGCTGCTTCCAAACC-3′, and mouse *IL-1β_ Reverse*: 5′- ATACTGCCTGCCTGAAGCTCTTGT-3′.

### LysoTracker staining

LysoTracker™ Deep Red (Invitrogen L12492, California, USA) was used to detect lysosomes. Dark incubation of cells with LysoTracker^™^ Deep Red at 37 °C was conducted for 30 min. At the end of incubation, wash three times with PBS and subsequently observe under a confocal microscope (Olympus, Tokyo, Japan).

### Dual luciferase assay

The Ctsb-3UTR-WT and Ctsb-3UTR-MUT (GYVB40032909) plasmids were constructed by Guangzhou Geneyuan Co., Ltd. (Guangzhou, China). Then, miR-223 NC and miR-223 OE cells were transfected with the plasmids. Finally, the firefly and kidney luciferase activity was detected using a dual luciferase reporter gene assay kit (Vazyme DL101-01, Nanjing, China).

### Western blot analysis

Spinal cords were collected and quantified with the BCA. Incubating 16 h at 4 °C with CTSB primary antibodies (Proteintech 12216-1-AP, Wuhan, China). A secondary antibody (Ray Antibody RM3002, Beijing, China) was applied for 1 h. The bands were visualized by a chemiluminescence kit (Affinity KF005, Jiangsu, China) and Image Lab software (Bio-Rad, Hercules, CA, USA).

### Statistical analyses

Data was analyzed using SPSS 20.0 (IBM, New York, USA) and GraphPad Prism 5 (GraphPad Software, California, USA). A one-way ANOVA with Bonferroni post-correction was used to test for differences between groups. This study considered a *P* value less than 0.05 to be statistically significant. At least three replicates were conducted in each experiment.

## Results

### 1. Accumulation of LDs in demyelinated state

**Fig. 1:**
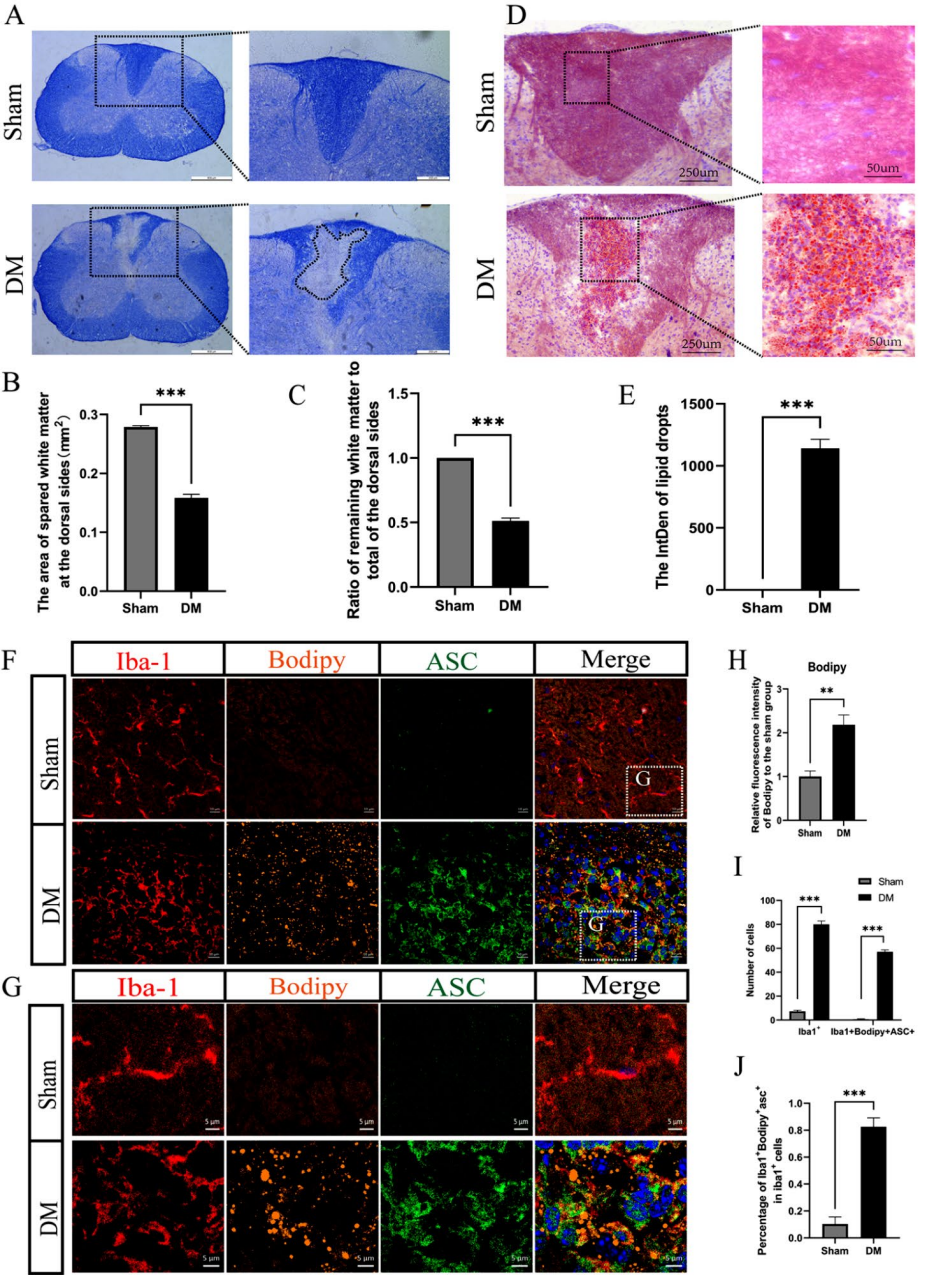
Accumulation of LDs in the LPC-induced demyelinated state. (**A**) EC staining of cross-sections of the injury center in the sham and demyelinated groups (scale bars = 500 μm). The lesion area was enlarged by 2.5 times and is displayed on the right side (scale bars = 200 μm). (**B**) Statistical analysis of the EC staining results showed that the area of residual white matter on the dorsal side of the spinal cord was significantly reduced after demyelination compared with that in the sham group. (**C**) Statistical analysis of the EC staining results showed that the ratio of the dorsal residual white matter to the total dorsal white matter was reduced after demyelination. (**D**) Oil Red O staining showing LD deposition in the spinal cord at the site of injury after demyelination (scale bars = 250 μm). The lesion area was enlarged 5-fold and is displayed on the right side (scale bars = 50 μm). (**E**) Statistical analysis of the results of LD staining revealed a significant increase in LDs in the dorsal white matter of the spinal cord after demyelination. (**F**) Immunofluorescence staining showing Iba-1 + microglia (red), BODIPY + LDs (yellow) and ASC+ (green) (scale bar = 10 μm). The dashed box shows the approximate area imaged in (**G**). (**G**) Immunofluorescence staining showing high-magnification images of Iba-1 + microglia (red), BODIPY + LDs (yellow) and ASC + microglia (green) (scale bar = 5 μm). (**H**) Immunofluorescence staining revealed that the number of LDs in microglia (Iba1 + BODIPY+) was significantly greater in demyelinated microglia than in sham-operated microglia. (**I**, **J**) The number of immunofluorescence-stained microglia (Iba1+) and Iba1 + BODIPY + ASC + cells and the proportion of Iba1 + BODIPY + ASC + cells to all microglia were determined. There were more microglia in the dorsal white matter of the spinal cord in the LPC-induced demyelination group than in the sham-operated group, and the Iba1 + BODIPY + ASC + cells and Iba1 + BODIPY + ASC + cells as a proportion of the microglia were increased. Sham: sham group; DM: demyelination group. All the data are presented as the mean ± SEM. *P < 0.05, **P < 0.01 and ***P < 0.001

To investigate the dynamics of demyelination, a two-point LPC-induced demyelination mouse model has been used in this study, which displays characteristic demyelinating lesions in the white matter of the spinal cord [25]. EC staining was used to identify lesion areas and subsequently quantify them. The staining results revealed pronounced white matter loss on the dorsal aspects (Fig. 1A). Quantitative analysis demonstrated a significant reduction in the area of preserved white matter in the dorsal regions in the demyelination (DM) group compared to the sham group (0.1583 ± 0.06 vs. 0.2790 ± 0.02 mm² (mean ± SEM); *P* < 0.001) (Fig. 1B). Moreover, a marked difference was noted between the sham and DM groups in the proportion of spared white matter relative to the total white matter on the dorsal sides (Fig. 1C). Following delineation of the lesion area, Oil Red O staining was utilized to identify LDs within the lesion zone (Fig. 1D). The results indicated a homogenous distribution of LDs, denoted by a uniform red hue in the sham group. In contrast, the DM group displayed a conspicuous accumulation of bright red LDs in the lesion area. Additionally, LD grayscale analysis revealed a substantial increase in LD accumulation in the lesion area following LPC-induced demyelination compared to that in the sham group (0.000 ± 0.00 vs. 1142 ± 72.45 (mean ± SEM); *P* < 0.001) (Fig. 1E).

LDAM was identified in the spinal cords of the demyelinated mice. To characterize the inflammatory state of microglia, the expression of ASC specks had been examined in this study, which are indicative of inflammation. By ecolabeling microglia/macrophage markers Iba-1 and ASC, the morphology and population of microglia within the dorsal white matter were identified. Microglia in the sham group exhibited extensive tertiary and quaternary branching, characterized by minimal overlap between branches. Conversely, microglia in demyelinated regions displayed pronounced cytosolic enlargement, a decrease in the length of protrusions, and a transition toward a rounded cellular morphology (Fig. 1F, G). Quantitative analysis revealed a significant increase in the number of microglia (Iba-1^+^) in the DM group (sham group 7.33 ± 0.882 vs. DM group 80.00 ± 2.887 (mean ± SEM)) (Fig. 1I). Subsequently, within the DM group, there was a notable accumulation of LDs in Iba-1-positive microglia, as evidenced by Iba1 + BODIPY + staining (sham group 1.00 ± 0.129 vs. DM group 2.18 ± 0.225 (mean ± SEM); *P* < 0.01) (Fig. 1F-H). This accumulation was accompanied by the upregulation of ASC expression in these LD-rich microglia (Iba1 + BODIPY + ASC+) (sham group 0.67 ± 0.333 vs. DM group 57.00 ± 1.732 (mean ± SEM)) (Fig. 1I), with a significantly greater proportion of BODIPY + ASC + in Iba1 + cells (sham group 0.10 ± 0.05 vs. DM group 0.83 ± 0.07 (mean ± SEM); *P* < 0.001) (Fig. 1J), indicating a pro-inflammatory state and the characteristic lipid droplet-accumulating microglia (LDAM) subtype. Collectively, these findings underscore the prevalence of the LDAM phenotype following demyelination, delineating a proinflammatory microglial subtype that may contribute to the pathophysiology of demyelination.

### 2. Enhanced lipophagy in the demyelinated state

**Fig. 2:**
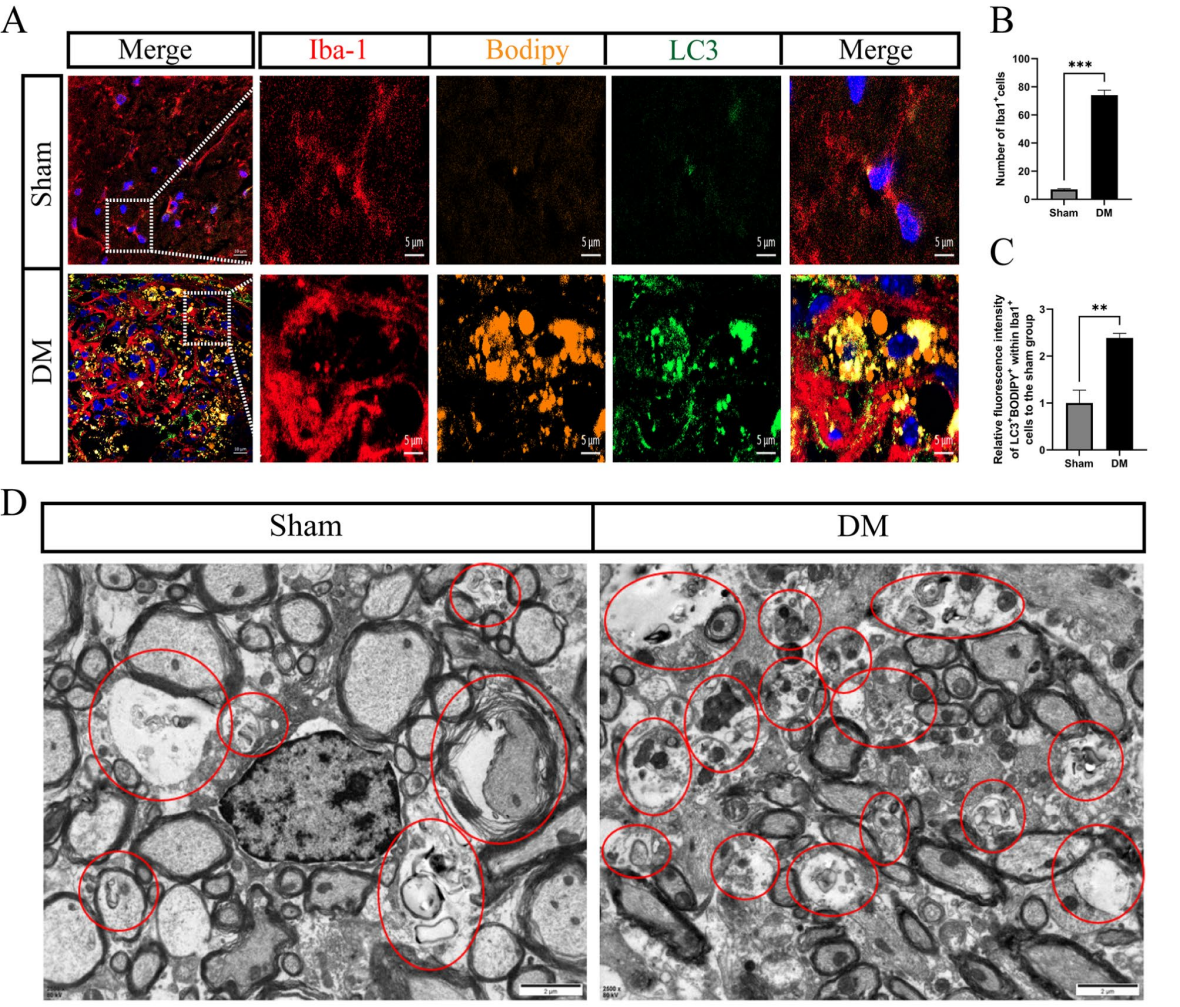
Enhanced Lipophagy Level in the Demyelination State. (**A**) The level of lipophagy (LC3 + BODIPY+) in microglia increased after demyelination (scale bars = 10 μm; scale bars = 5 μm (enlarged image scale in white box)). (**B**) The number of microglia (Iba1+) was determined by immunofluorescence microscopy, which revealed an increase after demyelination compared to that in the sham group. (**C**) The results of immunofluorescence staining for lipophagy (Iba1 + BODIPY + LC3+) in microglia were statistically significant, and the level of lipophagy in microglia was greater in the demyelination group than in the sham group. (**D**) TEM images of spinal cord tissue showing more autophagosomes (red circles) after demyelination (scale bars = 2 μm). Sham: sham group; DM: demyelination group. All the data are presented as the mean ± SEM. *P < 0.05, **P < 0.01 and ***P < 0.001

Lipophagy is a form of autophagy that selectively targets LDs for degradation [13]. To investigate whether microglia can degrade LDs through lipophagy in the lesion area, the colocalization studies were performed by using specific markers for autophagy (LC3) and fluorescent labeling of LDs (BODIPY) within microglia (Iba1+). Confocal microscopy revealed an increase in the number of Iba-1 + cells following demyelination (sham group 7.00 ± 0.577 vs. DM group 74.00 ± 3.606 (mean ± SEM); *P* < 0.001) (Fig. 2A, B), mirroring the findings in Fig. 1 in terms of cellular morphology. Furthermore, a significant amount of the LC3 protein was observed to associate with LDs within microglia (Fig. 2A). After quantification, the results revealed a significant increase in the fluorescence intensity of LC3 + BODIPY + within microglia in the DM group (sham group 1.00 ± 0.275 vs. DM group 2.39 ± 0.100 (mean ± SEM); *P* < 0.01) (Fig. 2C). Transmission electron microscopy (TEM) provided substantial evidence of elevated autophagic activity at sites of demyelination through the observed increase in lysosomes and autophagosomes (Fig. 2D). These findings suggest that after LPC-induced demyelination, microglia are capable of enhancing lipophagy levels, facilitating the degradation of LDs. Therefore, identifying the primary targets of microglial lipophagy could offer insights into the pathological accumulation of LDs.

### 3. Transcriptional analysis of mouse spinal cord tissue after demyelination

**Fig. 3:**
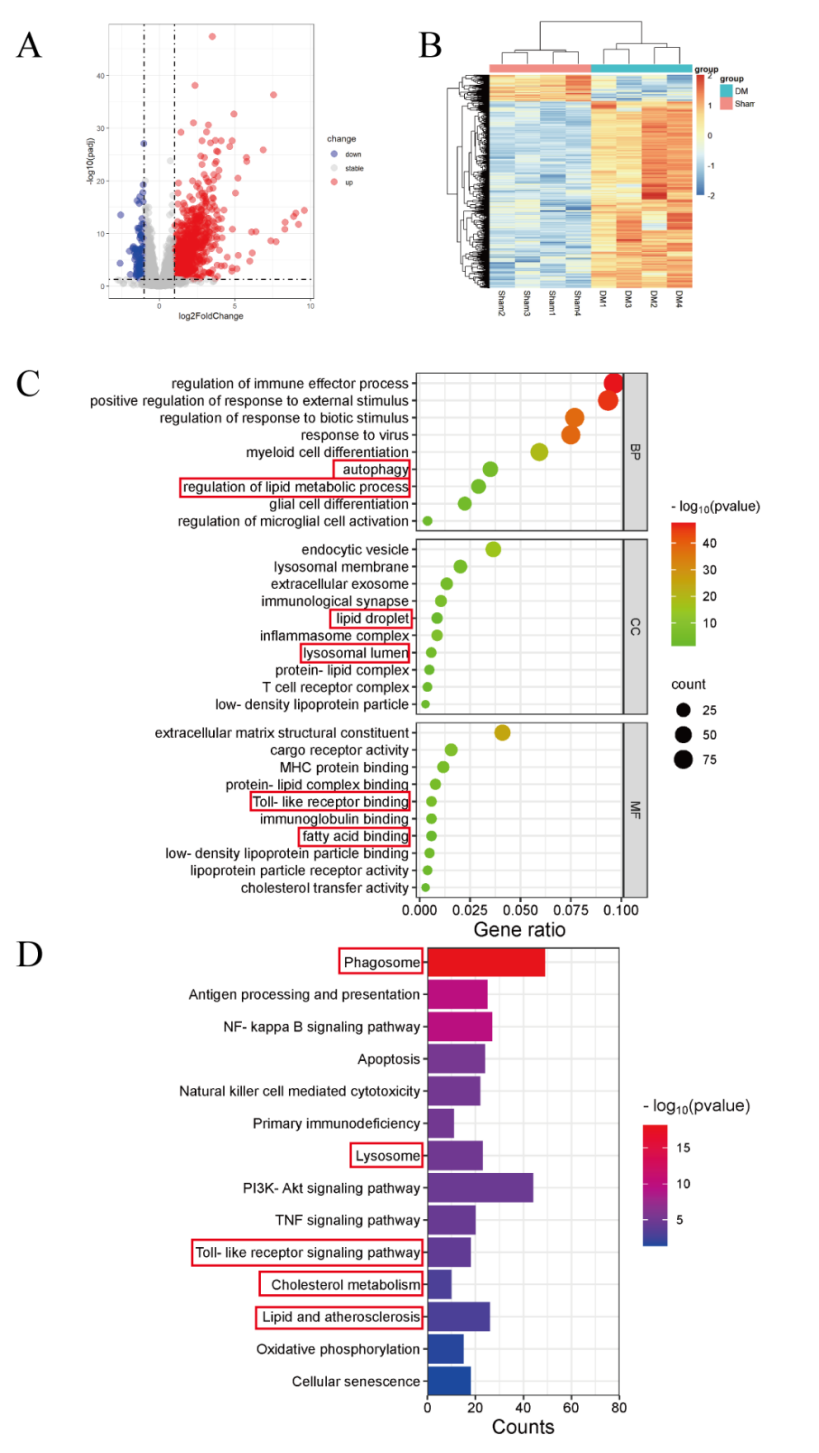
The microarray data and differentially expressed mRNAs in the microarray dataset were preprocessed. (**A**) Volcano plot of differentially expressed mRNAs; red indicates upregulated expression, gray indicates no difference in expression, and blue indicates downregulated expression; *n* = 4 per group. (**B**) Heatmap of differentially expressed mRNAs in the sham and DM groups, *n* = 4 per group. (**C**) Enrichment analysis of differentially expressed mRNAs in the microarray dataset. The bubble chart shows the Gene Ontology (GO) enrichment analysis of differentially expressed mRNAs. BP, biological process; CC, cellular component; MF, molecular function. (**D**) The bar plot shows the Kyoto Encyclopedia of Genes and Genomes (KEGG) analysis of differentially expressed mRNAs. Sham: sham group; DM: demyelination group. All the data are presented as the mean ± SEM. *P < 0.05, **P < 0.01 and ***P < 0.001

To investigate the molecular differences between demyelinated and sham spinal cord tissues, the transcriptomic profiles were analyzed from sham-operated mice and mice 14 days after LPC-induced demyelination. The thresholds for selecting differentially expressed genes (DEGs) were an FDR < 0.01 and a fold change ≥ 2. Volcano plots and K-means clustering with heatmaps provided an overview of all DEGs (Fig. 3A, B). After comparison with the sham group, 6090 DEGs were identified in the postinjury group, including 3251 upregulated and 2848 downregulated genes (Fig. 3A, B).

Biological process analysis indicated the involvement of the DEGs in the immune response, autophagy, lipid metabolism, glial cell differentiation, microglial activation, response to stimulus, and bone marrow cell differentiation (Fig. 3C). Cellular component analysis revealed that the DEGs mainly functioned in lysosomes, LDL particles, LDs, endocytic vesicles, inflammasomes, and immune synapses (Fig. 3C). Molecular function analysis highlighted that the DEGs were involved in an extracellular matrix structure, fatty acid binding, cargo receptor activity, and cholesterol transfer (Fig. 3C). The KEGG results also suggest that lipid metabolism and autophagy are key in demyelinating disease progression (Fig. 3D).

### 4. MiR-223 regulates lipophagy and lysosomal alterations

**Fig. 4:**
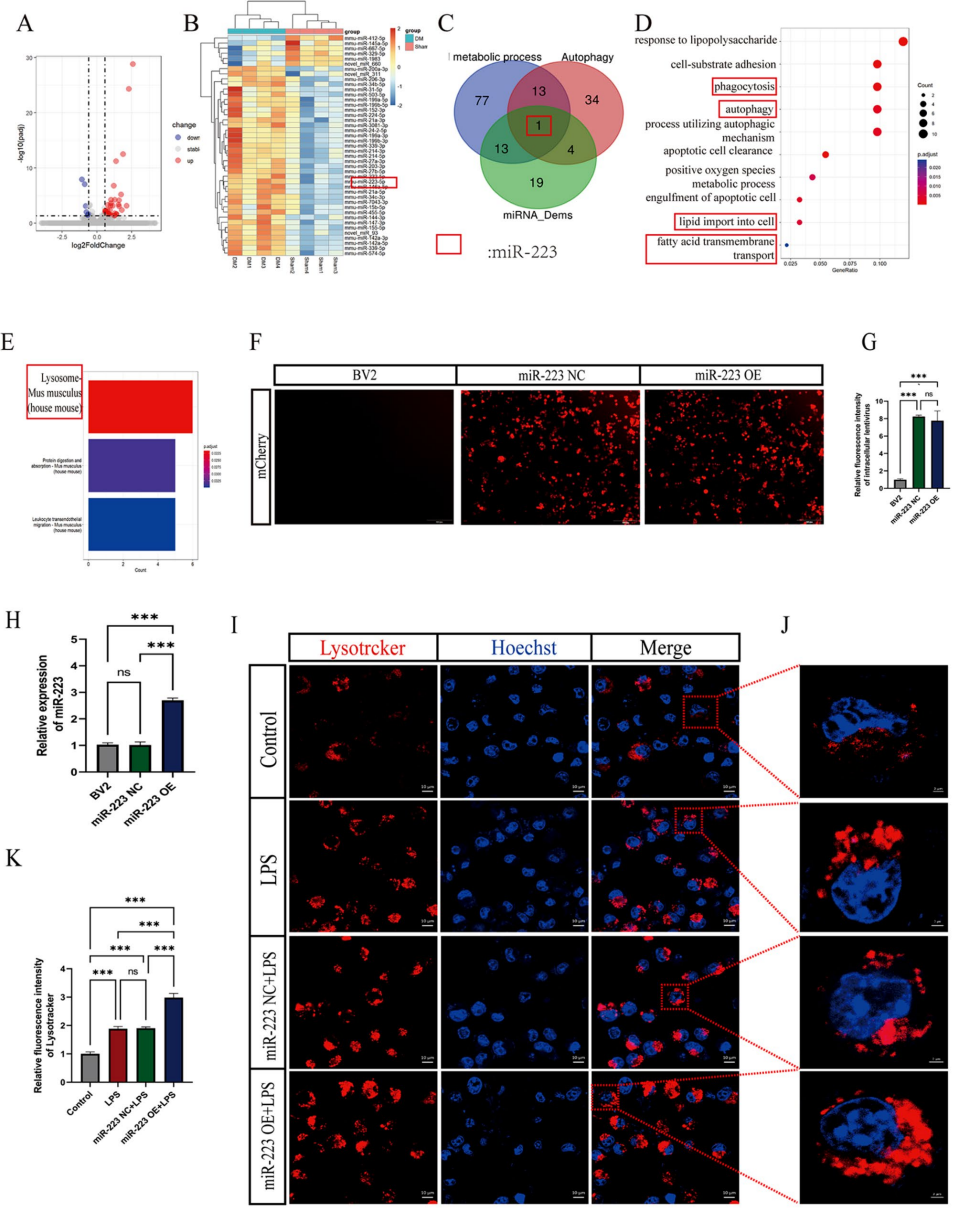
MiR-223 regulates lipophagy and lysosomal alterations. (**A**) Volcano plot of differentially expressed miRNAs; red indicates upregulated expression, and blue indicates downregulated expression. Gray indicates no difference in expression, *n* = 4 per group. (**B**) Heatmap of differentially expressed miRNAs in the sham and DM groups, *n* = 4 per group. (**C**) MiR-223: Lipid metabolism- and autophagy-related miRNAs in the demyelinated spinal cord group. Blue circles: lipid metabolism-related miRNAs; Red circles: autophagy-associated miRNAs. (**D**, **E**) GO and KEGG enrichment analyses of the genes common to both the miR-223 target genes and DM differentially expressed mRNAs. (**F**) Lentiviral autofluorescence (mCherry) was observed by inverted microscopy after lentiviral infection in each group of cells. (**G**) Quantitative analysis of intracellular fluorescence showed that the intracellular fluorescence of the miR-223 NC and miR-223 OE groups was significantly greater than that of the BV2 group. (**H**) Relative expression of miR-223 in each group of cells. There was no difference in the expression of miR-223 between the miR-223 NC group and the BV2 group; the expression in the miR-223 OE group was significantly greater than that in the miR-223 NC group and the BV2 group. (**I**) LysoTracker probe (Cy-5) was used to track the lysosomes of the cells in each group (scale bar = 10 μm). The dashed box shows the approximate imaging area of **J**. (**J**) Enlarged image of the LysoTracker probe (Cy-5) tracking the cytosol in each group (scale bar = 2 μm). (**K**) Quantitative analysis of the fluorescence intensity of the LysoTracker probe in the cells of each group. The fluorescence intensity of the LysoTracker probe in the miR-223 OE group was significantly greater than that in the other groups. miR-223 NC: empty lentivirus-infected BV2 cells; miR-223 OE: miR-223 overexpression lentivirus-infected BV2 cells; control: untreated BV2 cells; LPS: BV2 cells treated with LPS; miR-223 NC + LPS: miR-223 NC cells treated with LPS; miR-223 OE + LP: miR-223 OE cells treated with LPS. All the data are presented as the mean ± SEM. *P < 0.05, **P < 0.01 and ***P < 0.001

The criteria for selecting differentially expressed miRNAs (DEMs) were a *P* value < 0.01 and a fold change ≥ 2. The volcano plot (Fig. 4A) and K-means clustering with a heatmap (Fig. 4B) were used to visualize DEMs between the demyelinated and sham groups. Comparison with the sham group at 14 days postinjury, revealed that the expression levels of 43 miRNAs exhibited variations, showcasing 37 miRNAs with increased expression and 6 miRNAs displaying decreased expression. The DIANA mirPath v.4 database was used to identify miRNAs regulating autophagy and lipid metabolism pathways and overlapped them with the DEMs. The findings indicated the potential involvement of miR-223 in lipophagy. (Fig. 4C). Enrichment of target genes regulated by miR-223 and differentially expressed mRNAs indicated the association of miR-223 with autophagy, lipid transport, phagocytosis of apoptotic cells, and fatty acid transmembrane transport (Fig. 4D). These findings collectively support the hypothesis that miR-223 functions as a miRNA associated with lipophagy processes. In addition, miR-223 potentially induced lysosomal changes (Fig. 4E).

Lipophagy is closely associated with lysosomal function [13], therefore, the effects of miR-223 on lysosomal dynamics were verified. Lentiviral vectors were utilized to establish BV2 cell lines (miR-223 negative control cell line (miR-223 NC) and miR-223 overexpression cell line (miR-223 OE)). By observing and quantifying the lentiviral autofluorescence of mCherry, it was determined that the lentivirus had successfully infected the BV2 cells (Fig. 4F, G). Following that, the determination of miR-223’s relative expression ensued, and the results revealed a notably higher expression in the miR-223 overexpression (OE) group; moreover, there was no difference in the expression of miR-223 between the miR-223 NC group and BV2 cells (Fig. 4H). Then the cells were exposed to lipopolysaccharide (LPS) to simulate an inflammatory response, resulting in four distinct experimental conditions: control, LPS, miR-223 NC + LPS, and miR-223 OE + LPS. Observations of lysosomal changes were conducted after 24 h. Confocal microscopy analysis confirmed that the increase in miR-223 significantly enhanced lysosomal abundance (Fig. 4I, J), as evidenced by comparative quantification across the experimental groups (control vs. LPS vs. miR-223 NC + LPS vs. miR-223 OE + LPS: 1.00 ± 0.070 vs. 1.88 ± 0.083 vs. 1.91 ± 0.050 vs. 2.98 ± 0.143 (mean ± SEM), respectively) (Fig. 4K).

### 5. Identification of *Ctsb* as a direct target of miR-223-5p

**Fig. 5:**
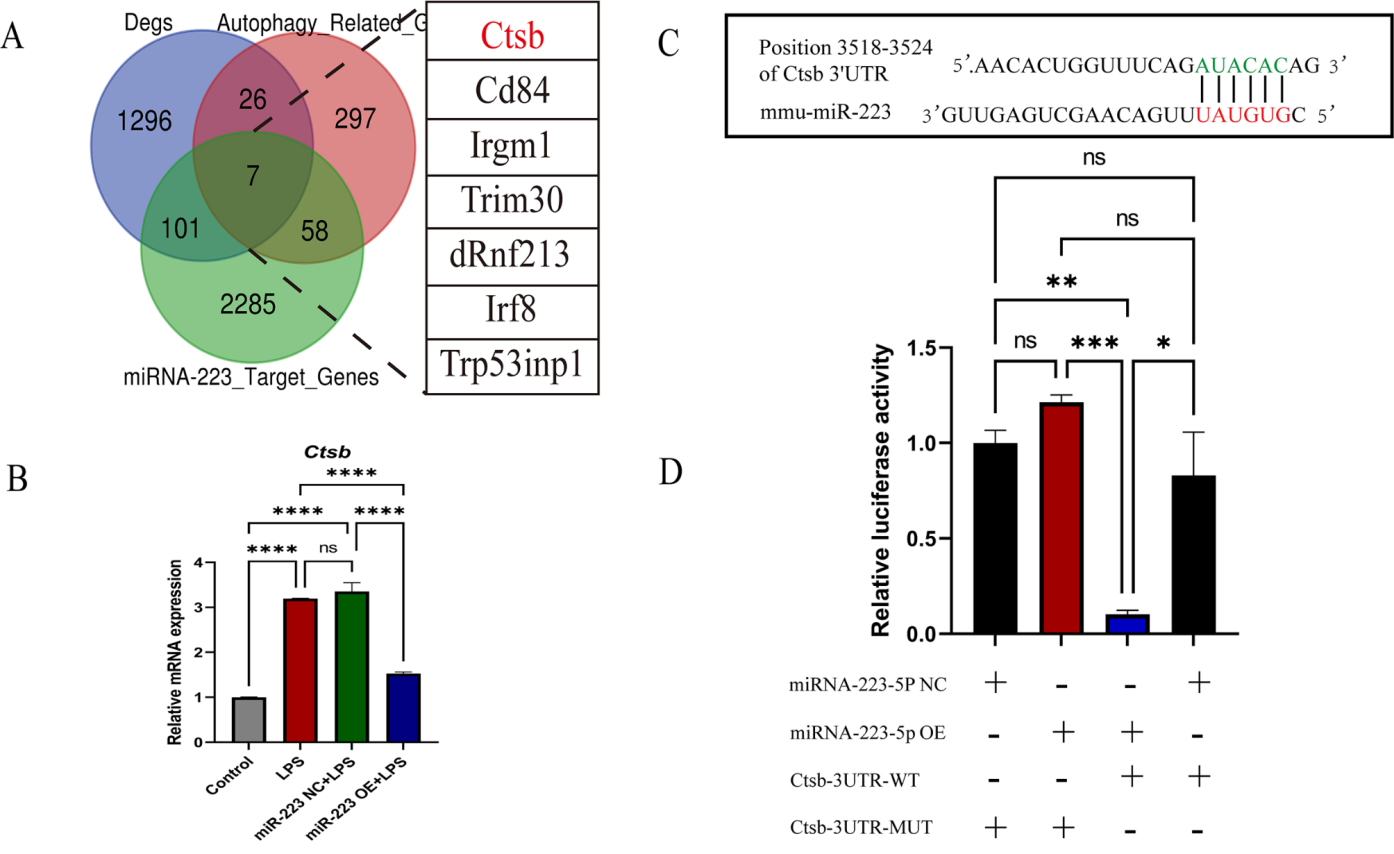
Identification of Ctsb as a direct target of miR-223-5p. (**A**) Prediction of autophagy-related miR-223 target gene candidates. (**B**) The q-PCR results showed that miR-223 regulates *Ctsb* expression at the posttranscriptional level. (**C**) The predicted target sequence of miR-223-5p in the 3′-UTR of *Ctsb*. (**D**) Luciferase activity in miR-223 NC and miR-223 OE cells transfected with the Ctsb-3UTR-wt or Ctsb-3UTR-mut reporter plasmid. Control: untreated BV2 cells; LPS: BV2 cells treated with LPS; miR-223 NC + LPS: miR-223 NC cells treated with LPS; miR-223 OE + LP: miR-223 OE cells treated with LPS. All the data are presented as the mean ± SEM. *P < 0.05, **P < 0.01, ***P < 0.001 and ****P < 0.0001

To elucidate the role of miR-223 in modulating lipophagy during demyelination, a comprehensive analysis was conducted by overlapping differentially expressed mRNAs, autophagy-related mRNAs, and predicted miR-223 target genes. The resulting overlap identified seven key genes, namely, *Ctsb*, *Irgm1*, *CD84*, *Trim30d*, *Rnf213*, *Irf8*, and *Trp53inp1*, as significantly enriched (Fig. 5A). Of particular interest was *Ctsb*, which is known for its dual roles as a lysosomal cathepsin and a regulator of autophagy and closely linked to the metabolism of lipids. This finding, coupled with the observed lysosomal alterations induced by miR-223 in BV2 cells, led us to hypothesize that *Ctsb* may serve as a critical target for miR-223 in the regulation of lipophagy.

Subsequent investigations aimed to determine whether miR-223 regulates *Ctsb* expression at the post-transcriptional level. The results confirmed this hypothesis: Overexpression of miR-223 significantly repressed *Ctsb* mRNA expression (control: 1.00 ± 0.008, LPS: 3.20 ± 0.005, miR-223 NC + LPS: 3.36 ± 0.192, and miR-223 OE + LPS: 1.527 ± 0.033 (mean ± SEM) (Fig. 5B). To further determine whether miR-223 downregulates *Ctsb* expression through binding to seed sequences, TargetScan was used to predict potential miR-223 binding sites within the 3′-UTR of *Ctsb* (Fig. 5C). Using the wild-type *Ctsb* reporter construct, luciferase activity was significantly decreased upon miR-223 overexpression as compared to the mutated construct (Fig. 5D). By reducing luciferase activity, miR-223 was demonstrated to directly interact with Ctsb mRNA, substantiating its role in post-transcriptional regulation.

### 6. MiR-223 inhibits CTSB and promotes lipophagy

**Fig. 6:**
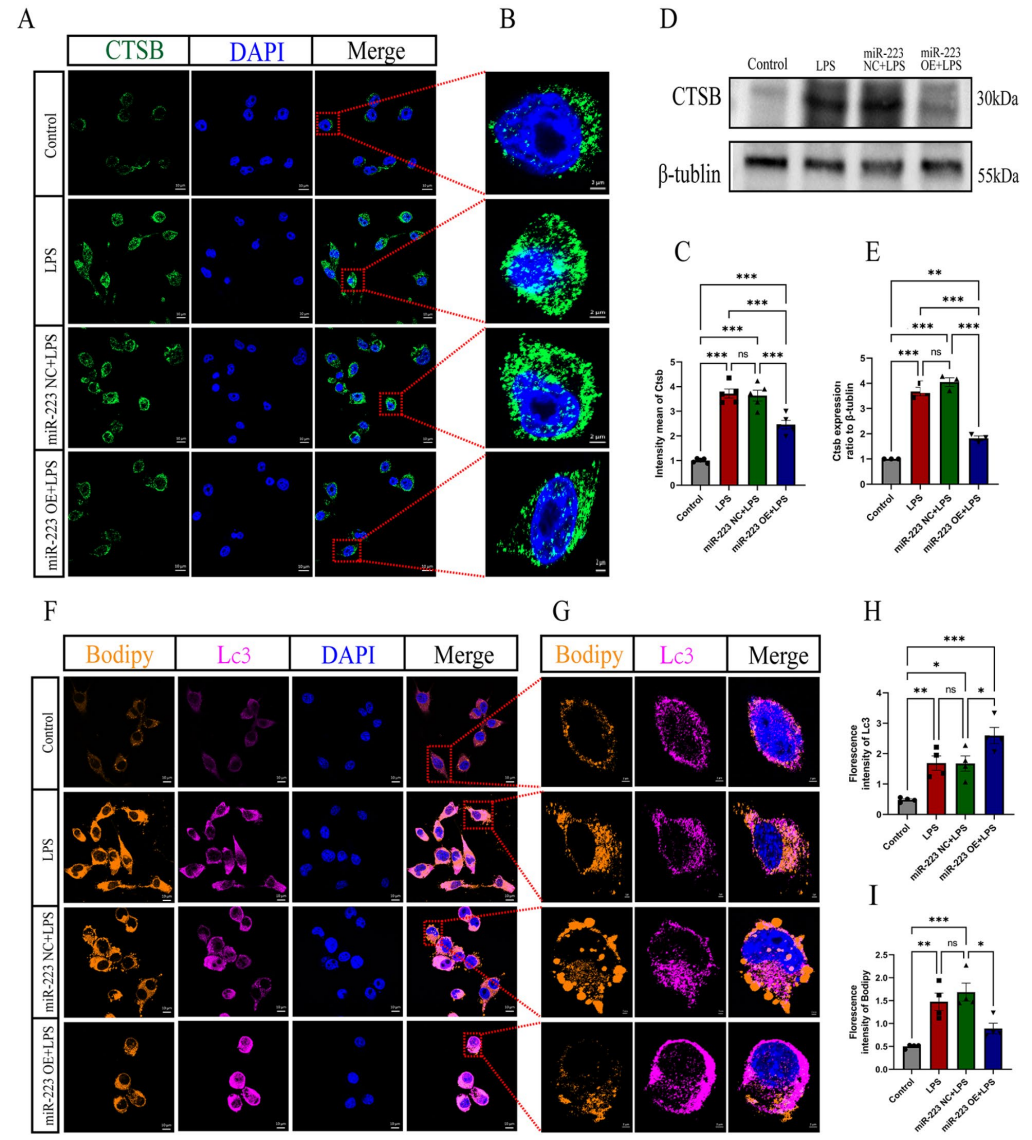
MiR-223 inhibits CTSB and promotes lipophagy. (**A**) Representative immunofluorescence images showing that the upregulation of miR-223 inhibited CTSB expression in BV2 cells (scale bar = 10 μm). (**B**) Partial magnification of Figure A (scale bar = 2 μm). (**C**) Quantitative CTSB fluorescence analysis showed that CTSB density in BV2 cells was significantly decreased after miR-223 upregulation. (**D**, **E**) Western blotting images and statistical analysis showed that miR-223 decreased the protein level of CTSB. (**F**, **G**) Immunofluorescence staining of spinal cord samples showing BODIPY + LDs (yellow), LC3 (pink), and DAPI (blue). (**H**) Quantification of LC3 + densities showed a significant increase in LC3 + densities in the miR-223 OE + LPS group. (**I**) BODIPY + density quantification showed that BODIPY + density decreased significantly in the miR-223 OE + LPS group. Control: untreated BV2 cells; LPS: BV2 cells treated with LPS; miR-223 NC + LPS: miR-223 NC cells treated with LPS; miR-223 OE + LP: miR-223 OE cells treated with LPS. All the data are presented as the mean ± SEM. *P < 0.05, **P < 0.01 and ***P < 0.001

Then the impact of miR-223 was explored on CTSB and lipophagy in BV2 cells. Following LPS stimulation, CTSB expression was assessed using immunofluorescence confocal microscopy. The results showed significantly lower CTSB expression in the miR-223 OE group than in the BV2 and miR-223 NC groups after LPS stimulation (1 ± 0.038 in the control group, 3.720 ± 0.182 in the LPS group, 3.639 ± 0.219 in the miR-223 NC + LPS group, and 2.459 ± 0.174 in the miR-223 OE + LPS group (mean ± SEM)) (Fig. 6A-C). Western blot analysis confirmed this trend (Fig. 6D, E). These results demonstrated the inhibitory effect of miR-223 on CTSB expression.

Furthermore, using LC3 and BODIPY fluorescence double staining, it was investigated whether miR-223 regulates selective autophagy in BV2 cells and its effect on LDs. Confocal imaging after LPS stimulation showed enhanced LC3 and BODIPY colocalization in BV2 cells, indicating increased lipophagy, with the miR-223 OE group showing an even greater effect (Fig. 6F, G). Statistical analysis revealed increased LC3 fluorescence intensity in the miR-223 OE + LPS group (0.96 ± 0.071 in the control group, 3.38 ± 0.469 in the LPS group, 3.35 ± 0.498 in the miR-223 NC + LPS group, and 5.19 ± 0.532 in the miR-223 OE + LPS group (mean ± SEM) (Fig. 6H) and a significant reduction in BODIPY fluorescence intensity in the miR-223 OE group (1.00 ± 0.045 in the control group, 2.95 ± 0.378 in the LPS group, 3.37 ± 0.394 in the miR-223 NC + LPS group, and 1.78 ± 0.230 in the miR-223 OE + LPS group (mean ± SEM)) (Fig. 6I). LC3 and BODIPY fluorescence intensities showed a negative correlation. These results highlight the role of miR-223 in enhancing LDAM lipophagy and accelerating intracellular LD degradation.

### 7. MiR-223 inhibits the expression of the inflammatory factor IL-1β

**Fig. 7:**
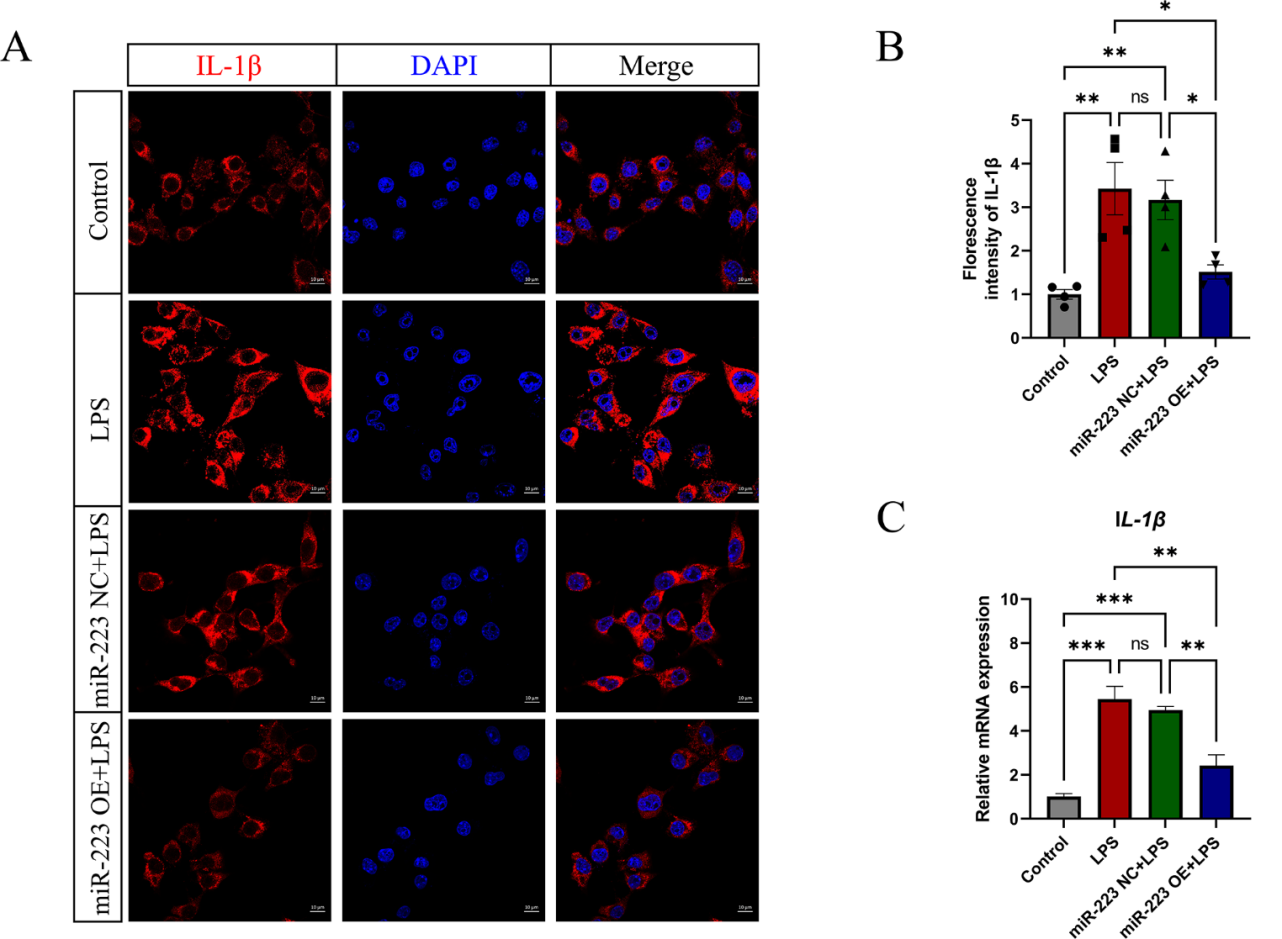
MiR-223 inhibits the expression of the inflammatory factor IL-1β. (**A**, **B**) Immunofluorescence images showing that the expression of the inflammatory factor IL-1β decreased after upregulation of miR-223. (**C**) q-PCR results showing that miR-223 decreased the mRNA level of IL-1β. Control: untreated BV2 cells; LPS: BV2 cells treated with LPS; miR-223 NC + LPS: miR-223 NC cells treated with LPS; miR-223 OE + LP: miR-223 OE cells treated with LPS. All the data are presented as the mean ± SEM. *P < 0.05, **P < 0.01 and ***P < 0.001

IL-1β is synthesized and secreted by a diverse array of immune and nonimmune cells upon exposure to inflammatory stimuli, and its expression is rapidly upregulated following LPS induction. Consequently, IL-1β concentrations serve as a barometer for the inflammatory state of immune cells. To elucidate the influence of miR-223 on the microglial inflammatory response, IL-1β expression levels across various experimental groups were assessed utilizing both immunofluorescence staining and q-PCR methodologies. Confocal imaging revealed increased IL-1β expression in control BV2 and miR-223 NC cells after LPS stimulation, while miR-223-overexpressing microglia exhibited decreased IL-1β levels (Fig. 7A). Statistical analyses provided robust evidence for the suppression of IL-1β following the overexpression of miR-223, with the measured values indicating a notable decrease (control group: 1 ± 0.1114, LPS group: 3.425 ± 0.6000, miR-223 NC + LPS group: 3.168 ± 0.4519, and miR-223 OE + LPS group: 1.515 ± 0.1619 (mean ± SEM)) (Fig. 7B). Similarly, q-PCR results mirrored this pattern, revealing a significant reduction in IL-1β expression (control group: 1.02 ± 0.130, LPS group: 5.46 ± 0.570, miR-223 NC + LPS group: 4.94 ± 0.16, and miR-223 OE + LPS group: 2.42 ± 0.496 (mean ± SEM)) (Fig. 7C). These findings collectively underscore the inhibitory impact of miR-223 overexpression on IL-1β production, consistent with the hypothesis that miR-223 plays a critical role in modulating the microglial inflammatory response. In summary, miR-223 appears to promote LD degradation by inhibiting CTSB, thereby enhancing lipophagy in LDAM and suppressing cellular inflammatory responses.

## Discussion

In this study, LDAM, a pathological cell subtype characterized by phagocytic defects and excessive LDs, was identified as prevalent in demyelinating lesions. Through a comprehensive multiomics analysis of miRNAs and mRNAs in demyelinated mouse spinal cord tissue, miR-223 was experimentally demonstrated as a novel regulator of lipophagy. The upregulation of miR-223 enhanced LPC-induced autophagy, which was coupled with a decrease in the number of LDs. Furthermore, CTSB was predicted as its target, and the experimental results revealed that the upregulation of miR-223 did inhibit the expression of CTSB and IL-1β in BV2 cells. In conclusion, it was demonstrated that the upregulation of miR-223 inhibits CTSB, promotes lipophagy-mediated LD degradation, and reduces the inflammatory response in microglia.

In recent years, the development of bioinformatics has accelerated progress in the study of human disease mechanisms. GO analysis revealed several terms related to demyelinating diseases, including regulation of immune response, microglial activation, and lipid metabolism. Microglia, the resident macrophages of the CNS [26], play a crucial role in clearing myelin debris from demyelinated sites for efficient remyelination and disease progression attenuation [27]. Initially, myelin-laden microglia exhibit a protective phenotype, characterized by the release of immunosuppressive and repair factors [28–30]. However, prolonged accumulation of myelin-derived lipids leads to the formation of numerous LDs, shifting microglia toward a phenotype that inhibits remyelination [5]. By inducing demyelinating injury in the mouse spinal cord, it was observed that the activation of numerous microglia laden with abundant LDs. The expression of ASC, which can regulate the activation of inflammasomes [31], was found to be high in LD-rich microglia at the site of demyelination, suggesting an obvious proinflammatory state. Other research groups have also identified LD-rich microglia in an aging mouse model. This subtype of microglia has phagocytic defects and secretes excessive proinflammatory cytokines, altering the immune microenvironment into a more inflammatory state [6]. Hence, this new subtype was named LDAM [6]. LD-rich microglia exhibit significantly reduced phagocytosis of myelin debris, indicating an association between increased damaged lipid storage and phagocytic defects [25]. Therefore, promoting LD degradation in LDAM at injury sites is crucial for restoring the phagocytic function and normal phenotype of microglia in treating demyelinating diseases.

Transcriptomic bioinformatics analyses have shown that autophagy is a key factor in demyelinating diseases. Histological staining of demyelinated spinal cord tissue revealed the presence of LDAM, along with enhanced autophagy in microglia at injury sites. Changes in microglial autophagy can regulate immune responses and neuroprotective effects [32–34], and prevent cellular senescence [35]. Enhanced autophagy has also been shown to reduce LD accumulation in foam cells [36]. As early as 2009, it was discovered that LD can be degraded through autophagy in a process known as lipophagy [11]. In this type of selective autophagy, LDs are targeted to lysosomes through ubiquitination and recruitment of specific lipid-selective autophagy factors and receptors [12, 14, 15]. Lipophagy regulates LD clearance and the efflux of stored cholesterol from macrophage foam cells through a process known as reverse cholesterol transport [37]. This discovery laid the groundwork for subsequent research. Similar to LDAM-rich MS, atherosclerosis is also characterized by lipid accumulation. Impaired macrophage autophagy is often associated with increased lipid accumulation [38] and excessive inflammasome activation in plaques [39, 40]. Conversely, enhancing macrophage autophagy and lysosomal biogenesis can reduce atherosclerosis [41]. In the nervous system, microglia lacking autophagy exhibit impaired myelin debris uptake and degradation [42], potentially due to autophagy deficiency inducing LD formation, which transforms microglia into phagocytically defective and inflammation-driven LDAM subtypes. Previous studies have indicated that promoting autophagy in a demyelinating state can accelerate LD degradation, activate lipid efflux systems, reduce the intracellular lipid load and inflammatory phenotypes of macrophages, and positively impact remyelination both ex vivo and in vivo [16]. These findings reveal the interplay between lipophagy and neuroinflammation, suggesting that inducing lipophagy holds great promise for improving remyelination in MS and offers a new approach to treating neurological diseases.

Using the DIANA mirPath v.4 database, the authors explored miRNAs related to the regulation of autophagy and lipid metabolism pathways. After overlapping these miRNAs with the differentially expressed miRNAs, the results identified miR-223 as a key regulator of lipophagy in demyelination. Researchers have previously identified miR-223 as a conserved anti-inflammatory microRNA, it is involved in numerous biological processes, including development, differentiation, hematopoiesis, and immune regulation [17, 43–46]. MiR-223 has been shown to regulate cholesterol metabolism and inflammatory signaling pathways by targeting cholesterol biosynthesis pathways, reversing foam cell formation in VSMC macrophages [47]. It can also activate the PI3K/AKT pathway to block TLR4 signaling and inhibit atherosclerosis development [48]. In the nervous system, miR-223 plays a crucial role in promoting the effective activation and phagocytosis of myelin debris by microglia, which are essential for initiating remyelination [49]. It facilitates microglial differentiation into an efficient M2 phenotype with enhanced phagocytic activity, inhibits NF-κB and the NLRP3 inflammasome, and regulates the immune microenvironment at lesion sites [47, 50, 51], aiding in repair while suppressing neuroinflammation. This discovery holds significant pathological and physiological relevance for multiple sclerosis and other neurodegenerative diseases. Nevertheless, the function of miR-223 in modulating lipophagy within the nervous system remains undocumented.

In vitro, LPS can induce BV2 cells to adopt the LDAM phenotype. A significant increase in LC3 expression and lysosome numbers was observed in LDAM. This phenomenon may be due to the following reason: during demyelination or LPS-induced acute inflammation, the autophagy-lysosome system in microglia is burdened with lipid digestion, leading to a lysosomal storage phenotype and pushing degradation and metabolism mechanisms to their limits. If lipid overload cannot be compensated by lysosomal digestion, substantial LD accumulation and the transformation of microglia to the LDAM subtype result. Hence, enhancing selective autophagy targeting LDs appears to be a feasible strategy for reversing the LDAM subtype. Fortunately, miR-223 can promote selective degradation of LDs by autophagy: the upregulation of miR-223 in LPS-stimulated BV2 cells enhanced intracellular autophagy levels and inhibited LD accumulation and IL-1β expression, thereby improving LDAM subtype transformation. It was indicated that miR-223 is an important regulatory factor in autophagy and lipid metabolism in other disease models [52–54] and the serum samples of ASO patients [54]. Overexpression of miR-223 in the VSMCs of ASO patients induces autophagy, significantly inhibiting foam cell formation and reducing intracellular total cholesterol levels [54]. Blocking autophagy with Atg7 siRNA was shown to weaken the inhibitory effect of miR-223 overexpression on foam cell formation, suggesting that miR-223 overexpression partially inhibits foam cell formation in VSMCs by inducing autophagy [54]. In this study, it was confirmed that miR-223 promotes lipid metabolism and inhibits the transformation of microglia into the LDAM subtype, improving the inflammatory response through lipophagy in microglia.

To identify the downstream target genes of miR-223 in lipophagy during demyelination, the miRNA, mRNA, and autophagy gene databases were analyzed collectively. The result showed that CTSB was a key target gene regulated by miR-223 in lipophagy. Previous studies have shown that cysteine proteases are crucial for protein hydrolysis and degradation in both lysosomal and extralysosomal environments and play indispensable roles in autophagy, antigen presentation, cellular stress signaling, metabolism, and lysosome-dependent cell death [55–57]. Whole-genome expression analysis has revealed that bone marrow-derived macrophages (BMDMs) lacking CTSB exhibit increased expression of TFEB, a central transcription factor that controls lysosomal and autophagy-related gene expression [26]. Transmission electron microscopy has revealed increased numbers and sizes of lysosomes and autophagosomes in CTSB-deficient BMDMs [56]. These results suggest that CTSB may inhibit the autophagy‒lysosome process under steady-state conditions. CTSB homeostasis is also critical for traumatic brain injury repair [58]. Bioinformatics analysis indicated that CTSB gene expression was upregulated in a demyelinated state, and a similar trend was observed in BV2 cells after LPS stimulation. It could be interpreted that the organism achieves relative homeostasis through feedback regulation., with negative feedback being more pronounced. Enhanced autophagy during demyelination or after LPS stimulation of microglia leads to upregulation of the negative regulatory factor CTSB to achieve negative feedback regulation of autophagy. Interestingly, miR-223 upregulation inhibited Ctsb, thereby promoting lipophagy.

### Strengths and limitations of the study

This study has several strengths. MiRNA and mRNA multi-omics analyses were utilized to determine the close relationship between miR-223 and lipophagy following demyelination, and Ctsb was identified as a potential target of miR-223 through database analysis. This is the advantage of the methodology. There are few studies on miRNAs related to lipophagy after demyelination, but the experimental results proved that the upregulation of miR-223 can promote lipophagy in microglia, which fills a theoretical gap in this area.

This study also has important limitations. First, due to the small size of LPC lesions, some intact tissue was present in the samples, potentially leading to a higher false-negative rate of differential genes and overlooking other key genes. Second, it was validated the miR-223-related pathway only in cellular experiments, which cannot fully simulate the complex physiological environment or directly reflect intricate interactions within organisms. Fortunately, other teams have demonstrated through in vivo experiments that miR-223 promotes the effective activation and phagocytosis of myelin debris by microglia [49]. Finally, the experimental design lacked a miR-223 knockdown or downregulated group. The aim was to study therapeutic factors post-demyelination, it was demonstrated that miR-223 has a positive effect in promoting autophagy and LD degradation; however, the opposite trend in microglia lacking miR-223 was not validated in this study.

## Conclusions

Microglia transformed into LDAM after inflammation-induced spinal cord demyelination. Overexpression of miR-223 effectively inhibited the inhibitory effect of CTSB on microglial autophagy, reducing LD accumulation and the release of inflammatory mediators. Thus, this study provides a new therapeutic idea for demyelinating diseases: inhibiting the inflammatory microenvironment by promoting lipophagy to suppress lipid droplets.

### Electronic supplementary material

Below is the link to the electronic supplementary material.

Supplementary Material 1

## Data Availability

Data is provided within the manuscript or supplementary information files.

## Declarations

Cell Communication and Signaling. This manuscript has not been presented.

elsewhere entirely.
